# Gender-Related Aspects in Osteoarthritis Development and Progression: A Review

**DOI:** 10.3390/ijms23052767

**Published:** 2022-03-02

**Authors:** Maria Peshkova, Alexey Lychagin, Marina Lipina, Berardo Di Matteo, Giuseppe Anzillotti, Flavio Ronzoni, Nastasia Kosheleva, Anastasia Shpichka, Valeriy Royuk, Victor Fomin, Eugene Kalinsky, Peter Timashev, Elizaveta Kon

**Affiliations:** 1 World-Class Research Center “Digital Biodesign and Personalized Healthcare”, Sechenov University, 119991 Moscow, Russia; peshkova_m_a@staff.sechenov.ru (M.P.); n_kosheleva@mail.ru (N.K.); ana-shpichka@yandex.ru (A.S.); timashev.peter@gmail.com (P.T.); 2 Institute for Regenerative Medicine, Sechenov University, 119991 Moscow, Russia; 3 Laboratory of Clinical Smart Nanotechnologies, Sechenov University, 119991 Moscow, Russia; marina.lipina@icloud.com (M.L.); eugene_kalinsky@mail.ru (E.K.); 4 Department of Traumatology, Orthopedics and Disaster Surgery, Sechenov University, 119991 Moscow, Russia; dr.lychagin@mail.ru (A.L.); berardo.dimatteo@gmail.com (B.D.M.); 5 IRCCS Humanitas Research Hospital, Rozzano, 20089 Milan, Italy; elizaveta.kon@humanitas.it; 6 Department of Biomedical Sciences, Humanitas University, Pieve Emanuele, 20090 Milan, Italy; flavio.ronzoni@hunimed.eu; 7 Human Anatomy Unit, Department of Public Health, Experimental and Forensic Medicine, University of Pavia, 27100 Pavia, Italy; 8 FSBSI Institute of General Pathology and Pathophysiology, 125315 Moscow, Russia; 9 Chemistry Department, Lomonosov Moscow State University, 119991 Moscow, Russia; 10 University Hospital No. 1, Sechenov University, 119991 Moscow, Russia; royuk_v_v@staff.sechenov.ru; 11 Department of Internal Medicine No. 1, Sechenov University, 119991 Moscow, Russia; fomin_vic@mail.ru

**Keywords:** osteoarthritis, cartilage, biomechanics, hormones, sexual dimorphism, sexual diversity

## Abstract

Osteoarthritis (OA) is a common degenerative joint disease treated mostly symptomatically before approaching its definitive treatment, joint arthroplasty. The rapidly growing prevalence of OA highlights the urgent need for a more efficient treatment strategy and boosts research into the mechanisms of OA incidence and progression. As a multifactorial disease, many aspects have been investigated as contributors to OA onset and progression. Differences in gender appear to play a role in the natural history of the disease, since female sex is known to increase the susceptibility to its development. The aim of the present review is to investigate the cues associated with gender by analyzing various hormonal, anatomical, molecular, and biomechanical parameters, as well as their differences between sexes. Our findings reveal the possible implications of gender in OA onset and progression and provide evidence for gaps in the current state of art, thus suggesting future research directions.

## 1. Introduction

Osteoarthritis (OA) is a degenerative joint disease and one of the leading causes of disability in elderly [1]. Affecting over 250 million people worldwide [2], its global prevalence is predicted to increase further owing to continuous population growth and increased life expectancy, accompanied by highly demanding functional outcomes [3,4]. The vast prevalence of this debilitating disease is reflected by the impacting economic burden on the global national healthcare systems, estimated to be up to 2.5 percent of the gross domestic product [3,5]. According to some predictions, the global economic impact of OA will double by 2030 [5], which legitimizes the huge research effort aimed at fully understanding the factors and all their possible implications in the disease onset and progression.

The disruption of the articular surfaces is only the most recognized macroscopic effects of the disease since all the joint tissues appear to be involved instead [6]. Degenerative changes in ligaments and meniscal tears are significant findings, but also Hoffa’s fat pad examination demonstrated increased vascularization and lymphocytic infiltration compared to healthy knees. Besides the soft tissues, the bone itself responds to the damage through MRI-visible signal alteration, defined as a ‘bone marrow lesion’, which is responsible for pain and considered a local expression of subchondral bone fat necrosis and marrow fibrosis [7,8,9].

Nowadays, the current state of the art on OA has extensively investigated the whole cohort of factors implied in OA development [2,5,6,10], including gender. The disease predilection towards females rather than males is a well-known fact [2,6,10]; however, the development of personalized medicine has drawn attention for the study of the exact mechanisms leading to these differences in disease prevalence and evolution. The complete understanding of the potential underlying mechanisms might help to propose a novel, articulated, and more efficient OA treatment, possibly leading the existing approaches towards a disease-modifying intervention rather than merely symptomatic treatment [5,11].

Women are known to be more prone to the development of OA [12]. The factors that might contribute to their susceptibility include thinner cartilage, tendency to varus malalignment, joint instability, and uneven mechanical loading. Furthermore, OA development can be triggered by the steep decline of sex hormone levels in the menopause [13,14]. Sex-related differences in the onset and progression of OA have been the subject of a number of studies, and there is increasing interest in this topic, with new data constantly brought to the attention of scientists [14,15]. Therefore, the present review aims to provide an updated summary of the most recent evidence on the gender-related mechanisms of OA, including the hormone-determined, anatomical, molecular, and biomechanical aspects, as well as their interconnections. The research will be focused on knee and hip OA, the two most common forms of the disease [5].

## 2. Search Methods

The extensive literature search was conducted via PubMed and Google Scholar with no date limitations applied. We used the following key words and their combinations: “osteoarthritis”, “cartilage”, “sex”, “gender”, “hormones”, “estrogens”, “testosterone”, “knee”, “hip”, “gait”, “biomechanics”, “men”, “women”, “males”, “females”, “molecular”, “biomarkers”. The last research was conducted on 31 January 2022, and two independent reviewers performed the research autonomously (MP, ML). We included only full-text articles specifically aimed at analyzing possible correlations between OA and gender. We included studies conducted in vitro, on animals, and on humans. We excluded editorials, letters, books, and abstracts, and restricted our search to English-language papers. Abstracts of the selected articles were screened, and full-text assessment was performed in case the data presented in the abstract were not sufficient or clear. Full-text assessment was also applied to all the reviews.

## 3. Results

In vitro, animal, and human studies have been included in this review. Based on this distinction, a synopsis of each study has been provided in Table 1, Table 2 and Table 3.

Based on the evidence from literature, OA can be regarded as a complex disease involving not only cartilage, but all the intra-articular and extra-articular tissues. In fact, beyond cartilaginous wear, it is possible to observe: (1) concurrent meniscal degeneration with the inherent loss of biomechanical properties; (2) synovial hypertrophy leading to an overall increase in the concentration of inflammatory cytokines and catabolic enzymes, such as metalloproteases; (3) subchondral bone plate disruption, characterized by the presence of fibrotic and osteonecrosis areas with lower mechanical resistance and inherent formation of osteophytes; (4) inflammation and fibrosis of the infra-patellar fat pad, which gradually loses its homeostatic role within the joint; (5) remodeling of peri-articular ligaments and tendons, which present an impaired ECM turnover leading to increased stiffness and mechanical insufficiency [7,8,9]. In the following sections, the impact of gender on all the articular tissues has been elucidated by considering the role of hormones and other molecular pathways and anatomical and biomechanical features.

### 3.1. Hormone-Determined Interactions

Studies specifically focused on hormonal-related aspects of OA onset and progression are presented, where the eventual differences between sexes are also highlighted (Table 4).

#### 3.1.1. Dehydroepiandrosterone (DHEA)

Dehydroepiandrosterone (DHEA) is the precursor of all sex steroid hormones, and its levels in both sexes is primarily age-dependent rather than sex-dependent. A steep drop of levels in DHEA and its sulfate, DHEAS, in the elderly has been clearly associated with the development of age-related conditions [45,46], therefore its role in the context of osteoarthritis has to be discussed. Notably, DHEA administration protocols used in many studies reported a successful cartilage protection effect in rabbit models of OA [20,23,24,25], especially in early [20] and middle-stage [20,21] OA.

DHEA is considered to exert its chondroprotective effects via several mechanisms, possibly implied first by the modulated expression of tissue remodeling factors, namely matrix metalloproteinases and their inhibitors MMPs/TIMP-1 [23,24], aggrecans and their inhibitors ADAMTS/TIMP-3 [25,47], and cysteine proteinases/cystatin C [20]. Another possibly implied mechanism is the suppression of proinflammatory cytokines, namely IL-1β [23,24]. There is also evidence that DHEA can regulate the *Wnt/β-catenin* signaling pathway, in which β-catenin levels appear to be essential for the maintenance of cartilage and myogenic homeostasis [16,48,49,50].

Li et al. suggested that the chondroprotective effect of DHEA on rabbit chondrocytes and cartilage [22] might be partly due to its aromatase-mediated conversion to estrogens by blocking both aromatase with letrozole and estrogen receptors with fulvestrant.

#### 3.1.2. Testosterone

Testosterone is the primary sex hormone in males and one of the precursors of both 5α-reduced androgens and estrogens.

The results of in vitro and in vivo studies of the effects of testosterone in OA progression are rather controversial. A study conducted by Koelling & Miosge revealed that physiologic concentrations of testosterone had a positive effect on the chondrogenic potential of chondrogenic progenitor cells (CPCs) in vitro [18]. On the other hand, Ma et al. demonstrated OA alleviation in orchiectomized mice in contrast to the control group, i.e., testosterone exacerbated OA in this model [17].

Testosterone was reported to have a positive association with the tibial cartilage volume in healthy middle-aged men [26]; however, at the same time, when studied longitudinally, higher serum free testosterone levels were associated with an increased rate of cartilage loss [27]. Hanna et al. speculated that this controversy may correspond to the indirect effect of testosterone on joints via increased musculoskeletal activity and therefore greater biomechanical loads, which lead to accelerated cartilage deterioration [27]. At the same time, they reported no correlation between testosterone levels and knee structure modification in middle-aged women without knee pain [28].

In contrast to the studies conducted in healthy men and women, Jin et al. focused on exploring the associations between endogenous sex hormones and joint structural changes in patients with symptomatic knee OA [29]. No statistically significant association was found when analyzing testosterone levels and their correlation with cartilage volume in all subjects regardless of sex; however, the group with the lowest serum testosterone levels was associated with increased knee effusion-synovitis and higher visual analogue scale (VAS) pain score in females, but not in males [29]. Similarly, De Kruijf et al. reported that low sex hormone levels are associated with greater chronic musculoskeletal pain in women [30]. At the same time, Freystaetter et al. reported that higher serum testosterone levels are associated with lower Western Ontario and McMaster Universities Osteoarthritis Index (WOMAC) pain scores in both men and women with severe knee OA, regardless of age, body mass index (BMI), and physical activity [31].

It was also suggested that the androgenic effects are dependent on the amount and activity of the 5α-reductase and aromatase enzymes, converting testosterone into dihydrotestosterone (DHT) and 17β-estradiol, respectively, as well as on the androgen receptor (AR) responses within the target tissues, rather than on serum free testosterone levels [28].

#### 3.1.3. Dihydrotestosterone (DHT)

Dihydrotestosterone (DHT) is one of the testosterone derivatives, and an even more potent androgen receptor agonist. In the study by Ma et al. discussed in the section above, DHT was demonstrated to restore OA severity in orchiectomized mice, who otherwise had less severe OA than the control group, i.e., DHT contributed to OA exacerbation along with testosterone [17].

#### 3.1.4. Estrogens and Progesterone

To date, increasing evidence suggests that estrogens play an important role in the maintenance of cartilage homeostasis [51], leading to the high prevalence of OA in postmenopausal women.

Premenopausal concentrations of estrogen in women were reported to promote the chondrogenic potential of CPCs in vitro [18]. Moreover, estrogens are reported to inhibit the MMP pathway in the cartilage [43], decreasing the amount of type II collagen degradation markers such as C-telopeptides (CTX-II) [44,52].

First, the gene expression of both estrogen receptor α and β (Erα, Erβ) was reported in human articular chondrocytes, with no significant difference between normal and osteoarthritic tissues [19]. However, the expression levels of both genes were significantly higher in men compared to women, suggesting a possible association with greater female susceptibility to estrogen level changes in the serum [19]. Regardless of joint size, *ERβ* was later reported to be highly represented in the synovial tissue in men and pre and postmenopausal women, while *ERα* amounts were reported to be variable in both sexes [53]. Moreover, the disruption of aromatase and estrogen synthesis is followed by an increase in ERα and ERβ expression in chondrocytes [54]. Villalvilla et al. emphasized that aromatase expression in the human cartilage is usually induced due to culturing. At the same time, compared to the bone and the synovium, in the native cartilage (both in healthy and OA), aromatase expression is almost undetectable. Therefore, cartilage should be considered as an estrogen target rather than a producer [55].

There is evidence that progesterone receptors (PR) are also expressed in chondrocytes and in chondroprogenitor cells, contributing to the regulation of their metabolism and affecting the subchondral bone structure. Due to uneven load distribution, changes in the subchondral bone architecture are known to affect the cartilage, thus promoting chondral wear and degradation [56]. Reportedly, low serum levels of both estradiol and progesterone are associated with increased knee effusion-synovitis in women, but not in men [29].

### 3.2. Molecular-Based Interactions

#### 3.2.1. Molecular Fundamentals in Pain Perception

Besides the different prevalence of OA in female sex, some recent studies have revealed than pain perception might be influenced by sex as well. A systematic review showed that women were more prone to suffer from more intense pain [57], which was in contrast to a more recent paper in which no statistically significant differences were identified [58]. However, even in a scenario of scarcity of consensus, it has been hypothesized that some mechanism related to pain perception may be more efficient in men rather than in women, as extensively proven by animal studies. One of the most relevant and recent reviews on the topic was conducted by Kim et al., and evidenced how significant differences in pain perception exist between male and females; the authors suggested how different molecular mechanisms, signaling pathways, and inflammatory cytokine expression could explain the variable pain perception in arthritis-induced pain in animal models [59].

A study conducted by Tsuda et al. revealed that the inhibition of spinal P2XRs reversed mechanical hypersensitivity in males but not in females [60]. Regarding inflammatory pain, it has been shown that phosphorylation of p38 is higher in males than females after tissue damage (both inflammatory or neuropathic), and that the inhibition of spinal p38 MAP kinase prevented pain only in male mice.

In addition, pain response appears to be different, as shown by Morales-Medina et al., who demonstrated reversion of mechanical allodynia by the administration of cerebrolysin, which appeared to be effective only in females [61].

Studies of humans also offered some important findings regarding pain perception differences between sexes: increased C-reactive protein and synovial fluid adiponectin were associated with more pain in females but not in males. In addition, plasmatic levels of leptin and adiponectin were associated with higher pain in females, while adipsin seemed to play a protective role; furthermore, resistin concentration was negatively correlated with pain intensity in men [32,33,62]. Thus, based on recent literature, OA is not only more prevalent in woman, but it seems to be more symptomatic due to a different molecular response to inflammatory stimuli.

#### 3.2.2. Biomarkers and Molecular Involvement in Disease Progression

Studies in animal models of OA evidenced how the disease molecular pathways are different in females and males. IL-6 gene knockout male mice developed more severe spontaneous OA, ECM catabolism, reduced proteoglycan synthesis, and increased subchondral sclerosis than females [63]. Furthermore, *Nov^del3^*^−/−^ male mice exhibited more cartilage destruction and subchondral sclerosis compared to females [6].

The hyaline cartilage of the involved joint is still considered the major target for clinical and molecular research in humans. A recent study conducted by Li et al. [15] demonstrated that cartilage has different gene expression between males and females, even in healthy joints: for example, *TSIX* transcript and *XIST* antisense RNA (*TSIX*) expression are significantly higher in females, although their function in the articular microenvironment is still under investigation. Moreover, most of the genes linked to OA onset are more involved in extracellular matrix turnover in females than in males. Interesting findings were also obtained when comparing the expression of OA cartilage genes between male and females. In profiling the genes expressed in OA, the molecular changes in females were significantly greater compared to males, possibly giving a further explanation for the severity of symptoms experienced by females. Among the upregulated and downregulated genes in OA, only *CISH*, *ADM*, *HLPDA*, *DDIT3*, *DDIT4*, *CFI*, *ST6GALNAC5*, *SPOCK1*, and *TNFSF15* showed similar expression in both genders, whilst the majority of the genes involved displayed significant gender-based differences. For instance, *FOXO*-mediated transcription factors are elevated in females’ osteoarthritic joints [34,64,65]. Conversely, in males, the *PERK-ATF4-CHOP* axis seems to be more involved in cartilage degeneration [15,66]. Male cartilage has also a lower expression of *IGFAL*, which encodes for a protein binding IGFs and increasing its half-life: therefore, the IGF-1 pathway seems to be less activated in males than in females [67,68].

In recent years, a huge effort has been conducted to investigate possible role of diagnostic biomarkers in OA and their difference prevalence in male and females.

Zhang et al. focused on searching for possible OA biomarkers shared by both genders; eight hub genes *POSTN*, *MMP2*, *CTSG*, *ELANE*, *COL3A1*, *MPO*, *COL1A1*, and *COL1A2* were proposed as possible biomarker for OA diagnosis [69].

Li et al. progressed even further, analyzing how collagen type I alpha 1 chain (*COL1A1*), collagen type I alpha 2 chain (*COL1A2*), matrix remodeling associated 5 (*MXRA5*), *THY1*, and TNF alpha induced protein 6 (*TNFAIP6*) have a higher response in females compared to males [70].

### 3.3. Anatomical & Biomechanical Cues

The following section aims to presenting the differences in anatomy of the joint, load distribution, and biomechanical variation that could possibly explain differences in OA incidence between males and females (Table 5).

#### 3.3.1. Anatomical Cues

The growing need for patient-specific prosthetic joint design has increased attention to gender-specific differences in the joint anatomy, but this is unfortunately still an extremely controversial topic. For example, it is known in the literature that the medial-lateral/anterior-posterior (ML/AP) aspect ratio in female knee joints is reduced; in other words, that female knee joints are smaller than the male joints [71,72,73,74]. Some researchers reported anterior condyles to be more prominent in men than in women [71], while others found no significant gender differences in this aspect, claiming that the condyle anatomy is highly variable regardless of sex [75,76]. Another controversial aspect of the knee joint anatomy is the Q angle, formed between the quadricep muscle and the patella tendon. There are reports showing that women have an increased Q angle [71] compared to men, as well as studies disproving this finding [75]. Many researchers claim that the differences observed in the anatomy of the knee joints are rather due to the patient’s morphotype than gender [73,75,77]. Moreover, there is no correlation between the aforementioned differences and the subsequent development of osteoarthritis.

Wise and colleagues aimed to determine whether the higher knee OA incidence in females was associated with specific bone shape via statistical shape modelling (SSM). Surprisingly, three out of thirteen modes (variations of bone shape) proposed by the researchers were reported to mitigate the risk of OA incidence in women. The authors reported the relative elevation and angle of the condyles to the shaft to be the crucial distinguishing features in these three models [35]. Another important finding is the difference in the knee joint congruity in males and females. It has been demonstrated that women present higher normalized contact area and lower congruity index values compared to men, confirming the higher risk of OA development [36].

Regarding gender-specific differences in hip joint anatomy, women tend to have a shorter femoral neck, a thinner femoral shaft, a lower femoral offset [78], a smaller femoral head diameter, and a greater anteversion of the femoral neck as well as greater acetabular anteversion and inclination [79]. The cervico-diaphyseal (CCD) angle or neck shaft angle is sometimes reported to be lower in women [78], while other researchers report no significant gender difference in this parameter [79]. Just as for the knee joint, the SSM approach was used to define OA-specific hip joint shapes; however, these studies did not address gender specificity [35] and therefore how the aforementioned anatomical characteristics could possibly contribute to OA development is yet to be defined. Still, some reports allow us to speculate on the association of these characteristics with OA. For instance, there is evidence that increased acetabular anteversion along with increased femoral anteversion can lead to poor congruity of the hip joint and therefore to OA development [80]. On the other hand, opinions differ on the contribution of neck shaft angle to OA development [81]: there is no evidence of correlation [82], also a lower angle can be considered as an OA risk factor, whilst an higher angle is regarded as a consequence of hip OA rather than a predisposing factor [83]. Hence, little and controversial data are available on gender differences related to joint anatomy associated with OA development, suggesting that the topic warrants further study.

**Table 5:** Associations between anatomical specificities and OA progression.

Parameter	Sex		Contribution to OA Development
	Women	Men	
Knee			
medial-lateral/anterior-posterior (ML/AP) aspect ratio (joint size)	smaller [71,72,73,74]	greater [71,72,73,74]	no correlation reported
anterior condyles	less prominent [71]	more prominent [71]	no correlation reported [71]
	no gender difference [75,76]		-
Q angle	greater [71]	smaller [71]	increased biomechanical stress in females [84]
	no gender difference [75]		-
normalized contact area	larger [36]	smaller [36]	poorer joint congruence in females [36]
congruity index	lower [36]	higher [36]	poorer joint congruence in females [36]
femoral neck	shorter [78]	longer [78]	no correlation reported
femoral shaft	thinner [78]	thicker [78]	no correlation reported
femoral offset	lower [78]	higher [78]	no correlation reported
femoral head diameter	smaller [79]	larger [79]	no correlation reported
acetabular inclination	increased [79]	decreased [79]	no correlation reported
femoral neck anteversion	increased [79]	decreased [79]	poorer joint congruence in females [80]
acetabular anteversion	increased [79]	decreased [79]	poorer joint congruence in females [80]
cervicodiaphyseal (CCD) angle or neck-shaft angle	lower [78]	higher [78]	stronger association with subsequent OA development in females [83]
			no correlation reported [82]
	no gender difference [79]		-

#### 3.3.2. Biomechanical Cues

Differences in joint anatomy obviously reflect biomechanical variations (Table 6). There is evidence that women tend to demonstrate increased hip flexion and reduced knee extension before initial contact, greater knee flexion moment in pre-swing, and greater peak mechanical joint power absorption at the knee pre-swing [37]. Kerrigan and colleagues point out the overall tendency toward higher peak joint powers and therefore greater mechanical work in female joints, as well as walking at higher cadences compared to males [37], leading to more intense cartilage wear and OA development.

The knee adduction moment (KAM) is one of the principal parameters assessed when analyzing the gait patterns. It characterizes the maximum medial knee load; in other words, the higher the KAM peak, the greater is the load on the medial compartment of the knee. Therefore, there is greater risk of developing OA in the knee medial compartment. There are controversial reports of women having lower [38] and higher [39] KAM compared to men; in addition, those parameters indicate that it is important to distinguish the first and the second KAM peaks. Only the latter is lower in women, while gender differences in the former are not significant [40]. Some authors propose the KAM impulse instead of KAM peak as a more comprehensive parameter of knee load since it takes into account load duration [85].

Load distribution is also associated with the knee alignment, since even a minimal increase in varus or valgus alignment significantly increases the medial or lateral tibiofemoral burden, respectively [86]. However, it seems that varus malalignment has a stronger association with OA development and progression [87,88], because even in neutrally aligned knees and regardless of gender, the overall load passing through the medial tibiofemoral compartment is greater than the one passing through the lateral compartment [89]. Interestingly, there is evidence that women experience dynamic deformation of lower limb bones toward varus as OA of the knee medial compartment progresses, contributing to OA severity [41].

## 4. Discussion and Future Perspectives

The knowledge of the multiple factors possibly implicated in OA incidence and development plays a crucial role in highlighting the direction for therapeutic treatment. To date, the well-known difference in gender must be fully evaluated when considering the prevalence of OA. The present review aims to collect the most relevant studies on the differences between genders, analyzing all the factors possibly involved in OA prevalence and development. Our main findings involve both hormonal and biomechanical aspects. When considering hormonal aspects, we found the correlation of the serum free high testosterone levels to both larger cartilage volume [26] and increased rate of cartilage loss [27] in males. This negative relation is possibly due to increased musculoskeletal activity since testosterone and its derivative, dihydrotestosterone (DHT), are known to increase physical function [90].

Surprisingly, it seems that low estrogens in postmenopausal women, known to be associated with OA development, might contribute to the disease progression for the opposite reason, namely increased muscle mass loss [91] and therefore muscle function impairment, which can lead to joint instability, uneven mechanical loading, and eventually to cartilage damage [42].

Estrogen deficiency not only activates muscle loss, but also bone loss. This process in postmenopausal women is very similar to the postpartum period, when a dramatic estrogen level drop is followed by osteoclast activation in order to provide sufficient blood calcium levels for lactation [92]. Although there is evidence that OA is associated with increased bone density rather than bone mass deficiency [93], in fact it seems that the former contributes to OA progression, while the latter triggers its initiation [94]. This process is also consistent with higher OA incidence in women. In general, while exerting direct effects on the articular cartilage, sex hormones have an even greater influence on OA development via their general effects on both muscles and bones.

The burden of anatomical and biomechanical cues appears to be also relevant when considering the different prevalence of OA in both sexes. In women, the anatomy is driven by the dynamic deformation of lower limbs towards varus which increases the load on the medial compartment, therefore contributing to cartilage loss (Table 5) [36]. Moreover, few aforementioned studies evaluated the impact of gait characteristics predisposing the female sex to greater mechanical work in joints (Table 6) [37,38].

At the gene expression level, OA onset and progression is sustained by different upregulation and downregulation of genes in males and females; even if the exact mechanisms involved are still unknown, the impaired extracellular matrix turnover seems to be more involved in cartilage degeneration in female patients [15].

Based on these finding, the different prevalence of OA can be justified not only by mere anatomical or biomechanical factors, but also by different molecular pathways activated between males and females [69,70].

Some notable preliminary findings can be garnered from this review; these findings reveal that the existing available literature focused on this aspect is still limited and often controversial, moreover evidencing the presence of a gap in the current knowledge.

To overcome this shortcoming, we propose a few insights, such as an enhanced patient stratification combined with innovative disease-modifying OA drugs (DMOADs) and cell-based therapies, which might result in the development of appropriate gender-specific therapies [95].

Additionally, the attention to time-related modifications in the disease progression (such as the transition from high bone turnover in early OA to decreased bone turnover in the late stages) or temporal variation in the pain type need accurate comprehension of the causal mechanistic alterations. In addition, the selection of proper medication for specific disease time points will help in defining gender-tailored treatments for each patient. Furthermore, it should be considered that OA may be characterized by combined endotype, such as an inflammatory pain endotype that could benefit from a combination of pharmaceuticals addressing both pain and inflammation [96,97].

Nevertheless, to date, mainly cartilage lesions have been treated with stem cell-based therapies, while tendons, subchondral bone, and other joint tissues were not considered for this. Since OA is acknowledged as a whole-joint disease, this is a central point to be considered [7,98].

Another crucial aspect to be accounted for future regenerative or pharmacological therapies is the mechanical status of the joint. It is important to address in a first-line therapy if the impaired joint mechanics triggering OA in both sexes. Consequently, if altered OA joint mechanics are not stabilized and biomechanical pathways are not reestablished, pharmacological or biological treatments might not be successful. Additionally, regulation of incorrect cellular mechanoreceptive pathways will give new chances to stop structural tissue weakening [99]. OA is not identified by a common pathophysiological pathway, since different pathways and risk factors are involved in the mechanical failure of the joint; thus, detecting early OA stages in both sexes will surely be helpful for the development of more effective and gender-targeted therapies. Hence, the identification of more specific biomarkers and innovative imaging techniques, as well as stronger interdisciplinary treatments, is required [100].

Gender-tailored OA therapy is a key point, and the current progresses in phenotype classification and targeted drug development will supply a pool of suitable therapeutic solutions in the future.

## 5. Conclusions

The reasons underlying the prevalence of OA in the female sex are not yet fully discerned. Estrogens have long been discussed in the context of OA progression in women. However, the role of other hormones in OA development in men and women has been poorly elucidated and warrants further study, since published data are often controversial.

In the field of biomechanics, despite some obvious differences between males and females, there is little evidence of the significant correlation between anatomical and mechanical specificities related to OA development. Molecular aspects and biomarkers will probably play a huge role in the future for the diagnosis and treatment of OA despite the insufficient clinical evidence so far. Moreover, some of these aspects remain debatable due to contradictory reports on the investigated gender differences. However, authors mostly agree that women tend to have poorer joint congruence and demonstrate greater mechanical work in the joints while walking, both factors that will promote cartilage wear. Understanding these differences in detail, both at the hormonal level and in regard to the anatomical and biomechanical characteristics of patients, could be helpful in the diagnosis and treatment of this degenerative joint disease and could further expand our understanding of its pathophysiology.

## Figures and Tables

**Table 1:** Synopsis of in vitro studies.

Study	Cells	Gender	Supplements	Assessment Targets	Assessment Methods	Major Findings
W. Li et al. [16]	Rabbit chondrocytes	F	A: DHEA B: DHEA + letrozole C: DHEA + fulvestrant D: DHEA + letrozole + fulvestrant	MMP-3, MMP-13, and TIMP-1 mRNA and protein levels	qPCR ELISA	The effects of DHEA are attenuated by the aromatase inhibitor letrozole and the estrogen receptor inhibitor fulvestrant The effects of DHEA may be mediated by its conversion to estradiol
Ma et al. [17]	Mouse chondrocytes	M and F	A: IL-1α + DHT B: IL-1α + E2	GAG	dimethylmethylene blue assay	Neither E2 nor DHT supplementation to male or female cartilage impacts the IL-1α-induced GAG release
Koelling & Miosge [18]	Human CPCs	M and F	A: Testosterone B: E2	Sox9 Runx2	ECLIA microarray analysis RT-PCR IHC Western blot	Physiologic concentrations of testosterone in men and premenopausal concentrations of estrogen in women have a positive effect on the chondrogenic potential of CPCs in vitro
Ushiyama et al. [19]	Human chondrocytes	M and F	N/A	ERα ERβ	RT-PCR	ER is expressed both in hip and knee chondrocytes, both in men and women, both in healthy and OA patients Expression levels of both genes are significantly higher in men than in women

qPCR—quantitative real time polymerase chain reaction; ELISA—enzyme-linked immunosorbent assay; CPCs—chondrogenic progenitor cells; ECLIA—electrochemiluminescence immunoassay; RT-PCR—real time reverse transcription–polymerase chain reaction; IHC—immunohistochemistry; ER—estrogen receptor.

**Table 2:** Synopsis of animal studies.

Ref	Sample Size/Model	Gender	OA Modelling	Assessment	Follow-Up	Major Findings
Bao et al. [20]	*n* = 108/rabbit *n* = 54: DHEA *n* = 54: control	M	ACLT	Histologic evaluation Gene expression	6, 9, and 12 weeks	Cysteine proteinases/cystatin C system and urokinase plasminogen activator/plasminogen activator inhibitor-1 system contribute to OA development DHEA suppresses both systems up to 9 weeks, but not up to 12 weeks DHEA exerts an anti-OA effect on the early stages of the disease
Huang et al. [21]	*n* = 30/rabbit *n* = 10: sham operation *n* = 15: DHEA *n* = 15: placebo	M	ACLT	Histologic evaluation	9 and 16 weeks	DHEA treatment delayed cartilage degeneration for up to 9 weeks DHEA treatment delayed cartilage degeneration for up to 16 weeks, but only in the lateral knee compartment DHEA can exert an anti-OA effect both on the early and middle stages of the disease
W. Li et al. [22]	*n* = 42/rabbit *n* = 12: sacrificed while the OA model establishment *n* = 6: DHEA *n* = 6: DHEA + letrozole *n* = 6: DHEA + fulvestrant *n* = 6: DHEA + letrozole + fulvestrant *n* = 6: DMSO (control)	N/A	ACLT	Histologic evaluation Gene expression	9 and 12 weeks	The effects of DHEA are attenuated by the aromatase inhibitor letrozole and the estrogen receptor inhibitor fulvestrant The effects of DHEA may be mediated by its conversion to estradiol
Jo et al. [23]	*n* = 22/rabbit *n* = 22: DHEA (right knee) *n* = 22: control (left knee)	N/A	ACLT	Histologic evaluation Gene expression	9 weeks	DHEA treatment delayed cartilage degeneration for up to 9 weeks: IL-1β 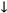 MMP-1 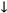 MMP-3 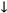 TIMP-1 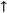
Wu et al. [24]	*n* = 40/rabbit *n* = 20: DHEA *n* = 20: control	N/A	ACLT	Histologic evaluation Gene expression	11 weeks	DHEA treatment delayed cartilage degeneration for up to 11 weeks: IL-1β 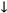 (in the synovium, but not in the cartilage) MMP-3 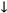 TIMP-1 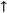
Huang et al. [25]	*n* = 10/rabbit *n* = 10: DHEA (one knee) *n* = 10: control (another knee)	M	ACLT	Gene expression	9 weeks	Aggrecanases 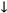 TIMP-3 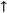
Ma et al. [17]	*n* = 139/mouse	M (intact and ORX) F (intact and OVX)	MMD	Histologic evaluation	8 weeks	Male mice had more severe OA than female mice Intact male mice had more severe OA than ORX mice, but the addition of DHT to ORX male mice re-established OA severity Intact female mice had more severe OA than OVX females, i.e., ovarian hormones decreased the severity of OA in female mice

ACLT—anterior cruciate ligament transection; MMP—matrix metalloproteinase; TIMP—tissue inhibitor of matrix metalloproteinase; MMD—medial meniscus destabilization; ORX—orchiectomized; OVX—ovariectomized.

**Table 3:** Synopsis of human studies.

Study	Sample Size/Gender	Healthy/OA	Assessment	Follow-Up (N/A in Cross-Sectional Studies)	Major Findings
Cicuttini et al. [26]	*n* = 45 (males)	healthy	Relationship between sex hormones levels and the tibial cartilage volume	N/A	Positive association of the serum testosterone level with total tibial cartilage and medial tibial cartilage volume
F. Hanna et al. [27]	*n* = 28 (males)	healthy	The factors determining cartilage loss	2 years	Positive association of the serum testosterone level with the tibial cartilage loss
F. S. Hanna et al. [28]	*n* = 139 (females)	healthy	Relationship between serum testosterone, preandrogens and SHBG levels, and the knee structure	N/A	Positive association between SHBG levels and patella bone volume
Jin et al. [29]	*n* = 200 males: 107 females: 93	OA	Relationship between endogenous sex hormones levels, the knee structure, and pain	2 years	Positive association of low serum endogenous estradiol, progesterone, and testosterone levels with increased knee effusion-synovitis in women
de Kruijf et al. [30]	*n* = 9811 males: 4266 females: 5545	healthy and OA	Relationship between sex hormone levels and chronic pain	5.6 ± 2.3 years	Positive association of low sex hormone levels and chronic musculoskeletal pain in women
Freystaetter et al. [31]	*n* = 272 males: 127 females: 145	OA	Relationship between testosterone level, knee pain, and function	N/A	Negative correlation of testosterone levels and pain in men and women Negative correlation of testosterone levels and disability in women
Calvet et al. [32]	*n* = 115 (females)	OA	Relationship between synovial fluid adipokines, pain, and function	N/A	Positive association of adiponectin and pain Positive association of resistin and disability Negative correlation of visfatin and disability
Perruccio et al. [33]	*n* = 87 males: 33 females: 45	OA	Relationship between plasma adipokine levels and pain	N/A	Positive association of leptin and adiponectin levels with pain in women Positive association of low adipsin levels with pain in women Negative correlation of resistin and pain in men
C. Li & Zheng [15]	*n* = 38 males: 22 females: 16	healthy and OA	Transcriptome dataset	N/A	Cartilage has different gene expression between males and females, even in healthy joints
F. S. Hanna et al. [34]	*n* = 271 males: 102 females: 169	healthy	Longitudinal gender differences in knee cartilage in a cohort of healthy adults	2.3 years	Greater annual percentage of total tibial cartilage volume loss in women Increased risk of tibiofemoral cartilage defects progression in women
Wise et al. [35]	*n* = 608 males: 229 females: 379	healthy and OA	Relationship between bone shape and OA incidence in men and women	N/A	Bone shape variations, namely the relative elevation and angle of the condyles to the shaft, can mitigate the risk of incident OA in women.
Tummala et al. [36]	*n* = 1595 males: 662 females: 933	healthy and OA	Gender differences in contact area and congruity index in the medial tibiofemoral joint	N/A	Higher normalized contact area and poorer congruence in women
Kerrigan et al. [37]	*n* = 99 males: 50 females: 49	healthy	Gender differences in joint biomechanics during walking	N/A	Increased hip flexion and reduced knee extension before initial contact, greater knee flexion moment in pre-swing, and greater peak mechanical joint power absorption at the knee pre-swing in women
Sims et al. [38]	*n* = 56 males: 26 females: 30	OA	Gender differences in joint biomechanics during walking in OA patients	N/A	Lower knee adduction moment and higher stride frequency in women
Ro et al. [39]	*n* = 84 males: 42 females: 42	healthy	Gender differences in joint biomechanics during walking in geriatric population	N/A	Higher peak KAM in women Increased mechanical loading on the knee associated with narrow step width and wide pelvis in women
Kumar et al. [40]	*n* = 76 males: 38 females: 38	healthy and OA	Gender differences in the knee cartilage composition and joint biomechanics in healthy and osteoarthritis populations	N/A	Higher lateral articular cartilage T1q and patellofemoral T1q in OA women Lower varus during walking in women Lower static varus and peak adduction moment in the second half of stance in middle-aged women Higher knee flexion moment in young women
Lu et al. [41]	*n* = 883 males: 199 females: 684	OA	Gender differences in the dynamic changes of lower limbs morphology in OA patients	1 month	Dynamic deformation of lower extremities and degeneration of articular cartilage in women, but not in men
Slemenda et al. [42]	*n* = 342 males: 164 females: 178	healthy and OA	Relationship between baseline lower extremity muscle weakness and incident radiographic knee OA	31.3 ± 10.0 months	Reduced quadriceps strength relative to body weight may be a risk factor for knee OA in women

SHBG—sex hormone binding globulin; ML/AP—medial–lateral/anterior–posterior; TKA—total knee arthroplasty; KAM—knee adduction moment.

**Table 4:** Highlights of hormonal-mediated differences between sexes.

DHEA	In vivo delays of knee cartilage degeneration for up to 9 weeks and up to 16 weeks, but only in the lateral knee compartment [21,23,24] Its effects seem to be mediated by the conversion to estradiol [22]
Estrogens	Dave a positive effect on the chondrogenic potential of CPCs in vitro [18] Decrease the severity of OA in female mice [43,44] Are negatively correlated with knee effusion-synovitis and chronic musculoskeletal pain in women [30]
Progesterone	Is negatively correlated with knee effusion-synovitis in women [29]
Testosterone	Has a positive effect on the chondrogenic potential of CPCs in vitro [18] Is negatively correlated with pain in men and women [29] Is positively correlated with total tibial cartilage and medial tibial cartilage volume and tibial cartilage loss in men [26,27] Is negatively correlated with knee effusion-synovitis, chronic musculoskeletal pain, and disability in women [29,30,31]

**Table 6:** Associations between gait characteristics and OA progression.

Parameter	Sex		Contribution to OA Development
	Women	Men	
Knee			
knee extension before initial contact	reduced [37]	increased [37]	greater mechanical work in joints; therefore, more intense cartilage wear in females [37,71]
knee flexion moment in pre-swing	increased [37]	reduced [37]	
peak mechanical joint power absorption at the knee pre-swing	increased [37]	reduced [37]	
knee adduction moment (KAM)	lower [38]	higher [38]	no correlation reported
	no gender difference in the first peak (KAM), second peak KAM lower in women [40]		no correlation reported
	higher [39]	lower [39]	greater knee medial compartment load in females [39]
hip flexion	increased [37]	decreased [37]	greater mechanical work in joints; therefore, more intense cartilage wear in females [37,71]

## Data Availability

All the data retrieved have been included in the final manuscript.

## Abbreviations

ADAMTS	a disintegrin and metalloproteinase with thrombospondin motifs
AR	androgen receptor
BMI	body mass index
CCD	cervicodiaphyseal
CPCs	chondrogenic progenitor cells
CTX-II	C-telopeptides of type II collagen
DHEA	dehydroepiandrosterone
DHEAS	dehydroepiandrosterone sulfate
DHT	dihydrotestosterone
ER	estrogen receptor
KAM	knee adduction moment
ML/AP	medial-lateral/anterior-posterior ratio
MMPs	matrix metalloproteinases
OA	osteoarthritis
PR	progesterone receptor
SSM	statistical shape modelling
TIMP	tissue inhibitor of metalloproteinase
VAS	Visual Analogue Scale
WOMAC	Western Ontario and McMaster Universities Osteoarthritis Index
